# A Finite Element Model of a MEMS-based Surface Acoustic Wave Hydrogen Sensor

**DOI:** 10.3390/s100201232

**Published:** 2010-02-02

**Authors:** Mohamed M. EL Gowini, Walied A. Moussa

**Affiliations:** Department of Mechanical Engineering, University of Alberta, Edmonton, AB, T6G 2G8, Canada; E-Mail: elgowini@ualberta.ca

**Keywords:** finite element analysis, Surface Acoustic Waves (SAW), hydrogen, piezoelectricity, MEMS, palladium

## Abstract

Hydrogen plays a significant role in various industrial applications, but careful handling and continuous monitoring are crucial since it is explosive when mixed with air. Surface Acoustic Wave (SAW) sensors provide desirable characteristics for hydrogen detection due to their small size, low fabrication cost, ease of integration and high sensitivity. In this paper a finite element model of a Surface Acoustic Wave sensor is developed using ANSYS12© and tested for hydrogen detection. The sensor consists of a YZ-lithium niobate substrate with interdigital electrodes (IDT) patterned on the surface. A thin palladium (Pd) film is added on the surface of the sensor due to its high affinity for hydrogen. With increased hydrogen absorption the palladium hydride structure undergoes a phase change due to the formation of the β-phase, which deteriorates the crystal structure. Therefore with increasing hydrogen concentration the stiffness and the density are significantly reduced. The values of the modulus of elasticity and the density at different hydrogen concentrations in palladium are utilized in the finite element model to determine the corresponding SAW sensor response. Results indicate that with increasing the hydrogen concentration the wave velocity decreases and the attenuation of the wave is reduced.

## 1. Introduction

Surface Acoustic Wave devices are considered to be the earliest types of MEMS due to the continuous electrical and mechanical interactions that take place during propagation. White and Voltmer [1] first reported the generation of Surface Acoustic Waves (SAW) on a quartz piezoelectric substrate. The waves were generated by applying a voltage signal to a set of finger-like electrodes patterned on the surface of a quartz substrate. This layout became known as the Delay Line structure. The SAW delay line offers an easy way of generating and detecting SAW on a piezoelectric substrate because the waves propagate along the free surface thus giving the user control over the signal, which can be sampled or modified according to the desired application. The delay line configuration is widely used in electronic devices such as radars to optimize the signal to noise ratio, pulse compression, band pass filters in TVs and as resonators. The confinement of the wave near the surface of the substrate allows it to be sensitive to changes in the external environment, therefore providing a plethora of sensing applications, which include detecting changes in mass, stiffness, viscosity, temperature, humidity, strain and force.

Hydrogen is widely used in industrial applications such as the preparation of ammonia and methanol, hydrogenation of organic compounds and in the production of semiconductors, petroleum recovery and refining, fueling spacecrafts and is used in fuel cells to power consumer electronic devices. Careful handling of hydrogen is crucial due to the various possible hazards. Diffusion of hydrogen into metals causes embrittlement, cracks and degradation in material properties which can cause catastrophic failure. In addition, hydrogen is explosive when mixed with air at a minimum ratio of 4% [2]. Due to its various applications and the possible hazards due to mishandling, careful monitoring of hydrogen leakage is crucial.

Various sensing mechanisms are adopted for hydrogen detection. D’Amico and Zemel [3] used pyroelectric sensors incorporating palladium electrodes for hydrogen detection. Hydrogen absorption causes heat generation, which affects the output voltage signal. Butler [4] used optical fiber sensors with palladium coated quartz fibers. Hydrogen absorption causes stretching of the optical fiber, which changes its optical length. Kumar [5] used an electrochemical cell where the potential difference between the two electrodes was used as an indicator of hydrogen detection. Cabrera [6] monitored the change in resistivity of thin Pd films due to hydrogen exposure at different hydrogen pressures. Resistivity increased during hydrogen exposure and then decreased to its initial value when hydrogen was removed from the chamber. In addition, it was found that if resistance measurements at a constant film temperature are made, the hydrogen concentration in the Pd film can be determined. Łukaszewski [7] used a Quartz Crystal Microbalance (QCM) for hydrogen detection. The QCM was coated with several films of pure Pd and Pd alloys. The frequency shift in each case was used as an indicator of hydrogen detection. QCM and SAW sensors have closely related operating principles, however SAW sensors offer increased sensitivity because they can operate at much higher frequencies [8]. As the operating frequency of SAW sensors increases the wave becomes more confined near the surface and therefore more sensitive to changes in the adjacent environment. There are various studies in the literature for the use of SAW technology for hydrogen detection [2,9–12].

**Figure 1.**
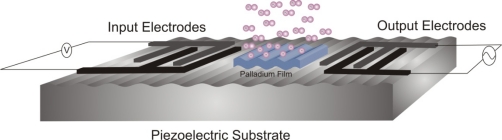
Layout of the SAW hydrogen sensor.

In this study a three dimensional Finite Element (FE) model of a SAW sensor is developed and tested for hydrogen detection. A chemically selective film is added on the surface of the sensor to absorb hydrogen and change the SAW properties accordingly. As illustrated in the above applications palladium is widely used for hydrogen detection, therefore in this study a thin palladium film is used as a chemically selective film. Figure 1 illustrates the layout of a SAW sensor adopting the delay line structure and covered with a palladium film.

Hydrogen has a high solubility in palladium which absorbs it like a sponge [13]. The hydrogen molecules break down in to atoms at the surface of the palladium film and then diffuse inside it to change the properties of the film with increasing concentrations. The change in wave velocity can be calculated from the change in phase of the frequency response. In addition, the insertion loss, which indicates the attenuation of the wave will be monitored at different levels of hydrogen absorption.

## 2. The Palladium Hydrogen System

Graham [14] observed that large volumes of hydrogen were absorbed or as he termed it *occluded* by palladium during electrolysis and since then the palladium hydrogen system has been one of the most experimentally investigated. Baranowski [15] showed that for different fcc metals and alloys the increase in the volume of the unit cell due to interstitial hydrogen is linear up to a concentration *c* = 0.75 a.f (atomic fraction). A hydride stoichiometry of *c* = 1 a.f can be achieved where the hydrogen atoms occupy all the octahedral sites of the Pd lattice, thus adopting an ideal sodium chloride structure [16]. When palladium absorbs (*n*) hydrogen atoms the change in volume (*V) is ΔV* = *nΔv*, where (*Δv*) is the change in volume per hydrogen atom. If the mean atomic volume of a palladium atom is (*Ω*), then the volume of Pd is *V* = *NΩ*. The relative volume change due to an atomic fraction c=nN is:

(1) ΔVV=c(ΔvΩ)

which is related to the lattice expansion approximately by:

(2) c(ΔvΩ)=3(Δaa)

where (*a*) is the lattice constant [16]. A wide collection of experiments that determine the relative volume change due to hydrogen absorption is available in Peisl [16]. Almost all of the experiments were carried out at room temperature and a value for the relative volume change ΔvΩ of 0.19 ± 0.01 was obtained. In this study the value of 0.19 for the relative volume change will be used.

At different hydrogen concentrations the corresponding density *ρ_c_* of palladium is calculated using equation (3) [17]:

(3) $$\rho_{c}=\left(\frac{1+\frac{m_{H}}{m_{\mathrm{Pd}}}\;c}{1+3\frac{\Delta a}{a}\;c}\right)\;\rho_{o}$$

where the molar masses *m_H_* and *m_Pd_* are 1.008 g/mol and 106.42 g/mol [18], respectively and *ρ_o_* is density of pure Pd.

In addition to a change in density, hydrogen absorption leads to changes in the elastic constants of palladium. The absorbed hydrogen atoms cause changes in the electronic structure of Pd, which have a direct affect on the frozen lattice component of the elastic modulus [17]. Absorbed hydrogen atoms occupy the interstitial octahedral locations in the lattice, hence displacing Pd atoms. This interaction results in a transfer of electrons and a change in the electron-to-atom ratio, which leads to an upward shift in the Fermi level and a reduction in the binding energy of *s* electrons due to lattice expansion [19,20]. Furthermore, significant temperature changes during hydrogen absorption leads to changes in the phonon components of the elastic modulus with increasing hydrogen concentration [17,20]. Finally, at low levels of hydrogen absorption the hydride gradient leads to precipitation hardening, which slightly increases the modulus of elasticity [20].

## 3. Background

### 3.1. Piezoelectricity

Hooke’s law is modified to include the electrical interaction that takes place in a piezoelectric material. There are various forms of the piezoelectric constitutive equations; the equations presented here are termed piezoelectric stress equations where the strain is an independent variable:

(4) $$T_{\mathrm{ij}}=c_{\mathrm{ijkl}}^{E}\;S_{\mathrm{kl}}-e_{\mathrm{ijk}}^{T}\;E_{k}$$

(5) $$D_{i}=\varepsilon_{\mathrm{ij}}^{S}\;E_{j}+e_{\mathrm{ikl}}\;S_{\mathrm{kl}}$$

*E_j_* is the electric field component and is measured in V/m; *D* is the electric displacement field, measured in C/m^2^; *ε_ij_* is the dielectric permittivity constant and is measured in F/m. The constants *e_ijk_* and *e_ikl_* are the piezoelectric stress constants (C/m^2^), which couple the electric and mechanical fields. The superscript (*T*) indicates that *e_ijk_* and *e_ikl_* are transposes of each other. The superscripts (*E*) on *ε_ij_* and (*S*) on *c_ijkl_* indicate that these are the properties at constant electric field and strain, respectively.

### 3.2. Wave Equations

Wave propagation in a piezoelectric crystal involves coupling of the particle displacement and the electric and magnetic fields. The equation of motion is coupled with Maxwell’s equations for electromagnetic fields through the piezoelectric constitutive equations, however this coupling is weak [21]. Since the solutions of interest are the acoustic waves, the magnetic field is assumed to be static and the electric field is calculated as the negative gradient of the potential:

(6) $$E_{i}=-\frac{\partial \phi }{{\partial r}_{i}}$$

This is the quasi-static approximation and has negligible effect on the solution [22]. By substituting the equation of motion into (Equation 4), expressing the strain in terms of the displacement components and utilizing the quasi-static approximation, the first piezoelectric constitutive equation can be re-written as:

(7) $$c_{\mathrm{ijkl}}^{E}\;\frac{\partial^{2}u_{k}}{{\partial r}_{j}{\partial r}_{l}}+e_{\mathrm{ijk}}\;\frac{\partial^{2}\phi }{{\partial r}_{k}{\partial r}_{j}}=\rho \frac{\partial^{2}u_{i}}{{\partial t}^{2}}$$

which is first wave equation.

Divergence of the second piezoelectric constitutive equation yields:

(8) $$\frac{{\partial D}_{i}}{{\partial r}_{i}}=\frac{\partial }{{\partial r}_{i}}\left(\varepsilon_{\mathrm{ij}}^{S}\cdot -\frac{\partial \phi }{{\partial r}_{j}}\right)+e_{\mathrm{ikl}}\;\frac{\partial^{2}u_{k}}{{\partial r}_{l}{\partial r}_{i}}$$

Piezoelectric materials are insulating materials thus ▿•*D* = 0 due to the absence of electric charge within the material. Therefore, the second wave equation is:

(9) $$\varepsilon_{\mathrm{ik}}^{S}\;\frac{\partial^{2}\phi }{{\partial r}_{i}{\partial r}_{k}}=e_{\mathrm{ikl}}\;\frac{\partial^{2}u_{k}}{{\partial r}_{i}{\partial r}_{l}}$$

Solving the wave equations yields three displacement equations and a fourth voltage equation, which are called partial wave solutions.

### 3.3. Inter-digital Transducers (IDT)

These are finger-like electrodes patterned on the surface of a piezoelectric substrate using lithographic techniques, as illustrated in Figure 1. They are used for launching and detecting acoustic waves on the surface of piezoelectric crystals. By applying a time varying voltage signal across the electrodes a mechanical wave is generated that propagates along the surface due to the converse piezoelectric effect. As the wave reaches the output IDT it is converted to a voltage signal due to the direct piezoelectric effect. Datta [23] provides detailed discussion on the operating principles of the different configurations of SAW-IDT devices, including the delay line structure.

## 4. Numerical Modeling using Finite Element Analysis (FEA)

Various numerical techniques have been used in modeling acoustic wave propagation in piezoelectric media; these include the Finite Element method (FEM), Finite Difference (FD) [24] and the Boundary Element Method (BEM). The FE method is the widely adopted technique especially for modeling the response of SAW sensors in 2D and 3D due to its versatility in modeling complex geometries for any set of material properties and loading conditions as long as the appropriate constitutive and equilibrium equations are satisfied [25–27]. In some instances a coupled BEM/FEM approach is adopted especially for modeling semi-infinite problems with periodic boundary conditions such as in SAW resonators [28]. The BEM adopts a periodic Green’s function for modeling the substrate and the FEM is used to model the electrodes with finite geometry and arbitrary shape [29].

The problem of interest in this study is to model the full device response using a 3D model of a SAW sensor; therefore the finite element method is adopted. A thorough discussion of the FE formulation for solving the wave equations in a piezoelectric medium has been published elsewhere [30].

### 4.1. Verification Model (ZnO-XY LiNbO_3_)

**Figure 2.**
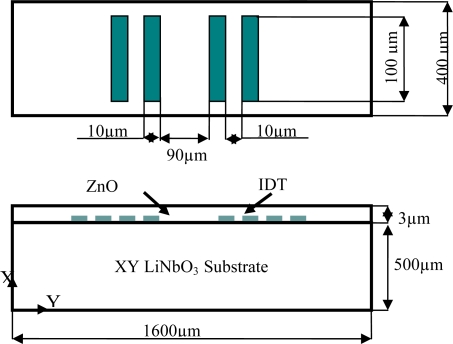
Schematic of the ZnO-XY LiNbO_3_ Layout.

Ippolito *et al.* [31] provides both simulation and experimental results for a specific configuration of a SAW sensor. The same configuration is adopted in this study for verification of the FE model. The device consists of a layered piezoelectric substrate with interdigital electrodes patterned on the interface. The piezoelectric substrate is lithium niobate (LiNbO_3_) with XY orientation, indicating that the X-crystal axis is perpendicular to the surface of the substrate, meanwhile the Y-crystal axis is along the propagation direction. The surface of the LiNbO_3_ substrate is completely covered with a thin piezoelectric zinc oxide (ZnO) film. Figure 2 illustrates a schematic of the device configuration with the corresponding dimensions.

Each of the input and output IDT’s on the surface consists of two electrode pairs. The electrode widths and spacing are equal and are 10 μm, thus the wavelength is 40 μm. The SAW velocity in this configuration is 4000 m/s [31]. The center frequency is calculated using:

(10) f=v/λ

which in this case is 100 MHz.

The material properties of lithium niobate are listed in Table 1. The density of lithium niobate is 4,647 kg/m^3^. The material properties are obtained from Wong [32].

The material properties of zinc oxide are listed in Table 2. The density of ZnO is 5,720 kg/m^3^. The material properties are obtained from Didenko [33].

Lithium niobate and zinc oxide are both piezoelectric materials and are meshed with the same element type. A coupled field element is selected, which has four degrees of freedom per node; displacements (*U_x_*), (*U_y_*), (*U_z_*) and voltage (*φ*). The elements at the ZnO-XY LiNbO_3_ interface are refined for accuracy. Mesh convergence studies show that an element size of 12 μm yields a 0.56% error.

The electrodes at the interface were modeled as a set of nodes coupled by a voltage degree of freedom (DOF). Xu [26] illustrated that at frequencies much less than 1 GHz the electrode mass has negligible effect on the frequency response of the SAW sensor. Various authors [13,34,35] have neglected the electrode mass to eliminate the second order effects and to reduce the size of the model.

**Figure 3.**
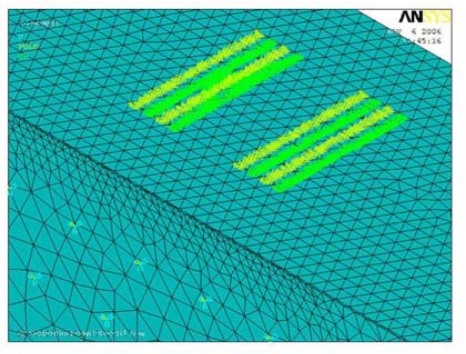
Electrodes modeled as coupled node sets.

Figure 3 illustrates the electrodes modeled as coupled node sets.

**Figure 4.**
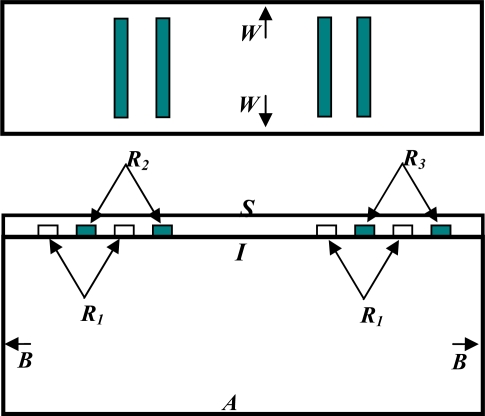
Boundary condition representation.

The boundary conditions on the model are listed below with reference to Figure 4:

- Clamped condition on the bottom surface **A**:
  (11) $$\left(u_{x},\;u_{y},\;u_{z},\;\phi =0\right)$$
- Continuity of the displacement field components *U_i_ for i* = *x*, *y*, *z* and the voltage *ϕ* at interface ***I***
- A Traction free boundary at the Free Surface ***S***:
  (12) $$T_{\mathrm{ix}}=0\;\;;\;\;\mathrm{for}\;i=x,\;y,\;z$$
- The following Dirichlet conditions for the electric potential:
  (13) $$\begin{array}{l} \phi {|}_{R1}=0\\ \phi {|}_{R2}=V(t)\\ \phi {|}_{R3}=O(t) \end{array}$$

where *V(t)* is the input voltage signal and *O(t)* is the output voltage.

Extension of the boundaries (*B*) along the length direction as indicated by the arrows and the same for the width boundaries (*W*). This condition is necessary to avoid wave reflections from the boundaries that would cause interference and hence deteriorate the response.

A transient analysis is carried out and the following impulse signal is applied at the input electrodes:

(14) $$V_{\mathrm{in}}=\{\begin{array}{lll} 1x10^{9} & \mathrm{for} & 0<t\leq T_{s}\\ 0 & \mathrm{for} & t>T_{s} \end{array}$$

where T_s_ is the time step size set to 1 ns. The simulation time is 100 ns. The frequency response is determined from the Fourier transform of the impulse response. Table 3 lists the results of the current simulation in comparison with the results from Ippolito *et al.* [31]. As can be clearly seen, results of the current simulation agree very well with both the simulation and experimental values. In the current simulation the center frequency value is 100.56 MHz, however the center frequency in the experimental result is 103 MHz. The 3 MHz variation is due to the tolerance involved in fabricating the electrodes using lithographic techniques. The insertion loss value in the current simulation is −35.5 dB, which more closely matches the experimental value of −34.3 dB than the simulation value of −37.5 dB by Ippolito *et al*.

Now that the model is verified the sensor configuration is altered to allow for hydrogen detection.

## 5. Finite Element Model of a SAW Sensor for Hydrogen Detection

**Figure 5.**
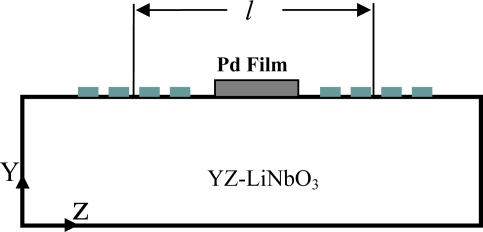
SAW sensor configuration for hydrogen detection.

In this configuration a bare YZ-LiNbO_3_ substrate is used and a thin palladium layer is added between the two sets of IDT’s as shown in Figure 5.

The YZ-LiNbO_3_ configuration is widely used for sensing applications due to its high SAW velocity and high electromechanical coupling coefficient [36]. This configuration has been used by various authors for hydrogen detection [9,11,12].

It has been illustrated that the density and elastic constants of the palladium film change with hydrogen absorption. The changing properties influence the phase velocity (*v*) of the wave, which is dependent on the material properties of the medium in which it propagates. For an isotropic medium:

(15) $$v=\sqrt{\frac{c}{\rho }}$$

where *c* is the stiffness constant (N/m^2^) and *ρ* is the density (kg/m^3^) of the medium. The change in wave velocity is used to evaluate the sensor response to hydrogen absorption. The change in velocity is calculated from the change in phase response of the sensor. The phase is defined as:

(16) $$\phi =-\omega \cdot T_{o}=-\omega \cdot \frac{1}{v}$$

where *ω* is the angular frequency in rad/s, *T_o_* is the time it takes the wave to travel a given distance (*l*) and (*v*) is the phase velocity of the wave in m/s. The change in phase is related to the change in velocity as follows:

(17) $$\Delta \phi =\omega \cdot \frac{1}{v^{2}}\Delta v$$

therefore:

(18) $$\left|\frac{\Delta \phi }{\phi }\right|=\left|\frac{\Delta v}{v}\right|$$

The change in phase is calculated with respect to the pure Pd case.

### 5.1. Finite Element Analysis of the YZ-LiNbO_3_ SAW Sensor Model

The material properties of lithium niobate are listed in Table 1; however the local coordinate system is rotated to obtain the YZ orientation. The lithium niobate substrate is meshed with a coupled field solid element with four DOF per node; displacements (*U_x_*), (*U_y_*), (*U_z_*) and voltage (*φ*). The boundary conditions for this model are slightly different than those for the ZnO-XY LiNbO_3_ layout. Continuity of the displacement field is imposed at the film-substrate interface and a stress free boundary condition is applied at the free surface of the Pd film. A stress free boundary is also applied to the LiNbO_3_ substrate surface. In addition, the following conditions apply;

- Clamped condition on the bottom of the substrate
- Same Dirichlet conditions for the electric potential at the electrodes
- Extension of the boundaries along the length and width of the substrate to avoid reflection

In deciding the operating frequency the goal is to increase the frequency level because at higher frequencies the wave becomes more confined near the surface and therefore more sensitive to changes in the adjacent environment. However, at higher frequencies the wavelength decreases and the size of the elements at the surface have to decrease leading to a more computationally expensive model. Various frequency levels have been attempted with the model size and simulation run time as constraints. A center frequency of 128 MHz is found to be an acceptable compromise. The parameters of the sensor are listed in Table 4.

#### 5.1.1. Palladium Film

The palladium film is meshed with a structural field element, which adopts three degrees of freedom per node; (*U_x_*), (*U_y_*) and (*U_z_*). The thickness of the Pd films is set to 2 μm, which is the minimum thickness that could be attained while maintaining a sufficient number of elements for increased accuracy. In addition, this thickness is reasonable and can be attained practically with common deposition techniques such as electron-beam evaporation.

The increase in volume of the Pd film due to hydrogen absorption is represented by an increase in the thickness direction, therefore neglecting the shear effects at the film-substrate interface. The Pd free surface can expand easily as it is an unconstrained surface. Film thickness values adopted in the simulations are listed in Table 5 and are determined using (Equation 1) and (Equation 2). Density of pure Pd is 12,020 kg/m^3^ [17] and the values at different hydrogen concentrations are calculated using (Equation 3).

In addition, the effects on the elastic constants are included by adopting the corresponding absolute values of the modulus of elasticity for different hydrogen concentrations, which are obtained from Fabre [17]. Material properties for the Pd film at different hydrogen concentrations are listed in Table 6. The Poisson’s ratio for Pd can be assumed to be invariant with concentration and the value for pure Pd (ν = 0.375) is maintained [37].

## 6. Results

**Figure 6.**
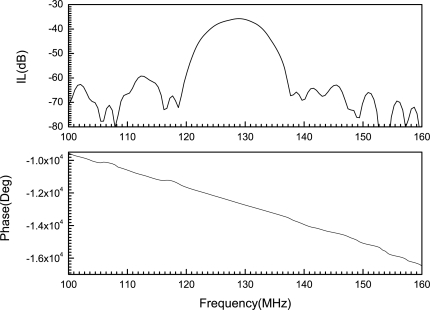
Frequency response for the pure Pd case, *f_c_* = 128 MHz.

A transient analysis is carried out to determine the sensor response due to the different levels of hydrogen absorption. The impulse signal in (Equation 14) is applied and the simulation is run for 400 ns. The frequency response is obtained from the time domain response by Fourier transform. The frequency response of the SAW sensor with a pure Pd film *i.e.,* no hydrogen absorption is shown in Figure 6.

**Figure 7.**
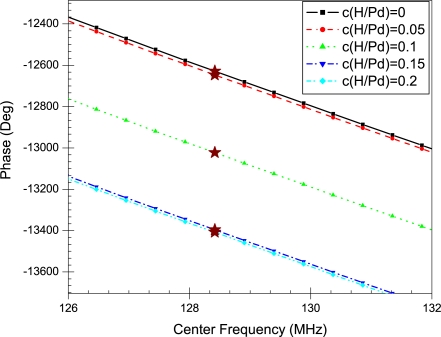
Phase profiles up to a concentration level of 0.2 a.f. The (⋆) sign illustrates the phase value at the center frequency.

**Figure 8.**
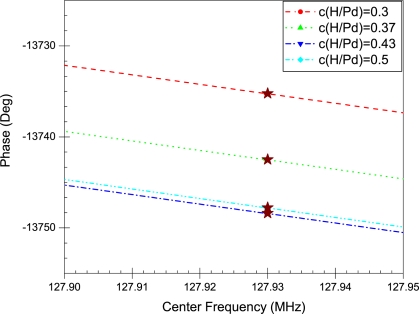
Phase Profiles for the concentration range 0.3–0.5 a.f. The (⋆) sign illustrates the phase value at the center frequency.

**Figure 9.**
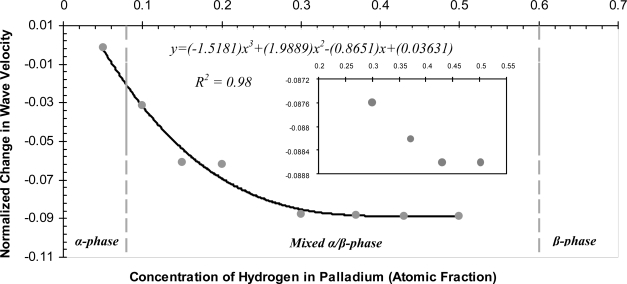
Normalized Change in Phase Velocity at Different Levels of Hydrogen Concentrations. R^2^ for Cubic Polynomial fit is 0.9848. Inset illustrates the Normalized Change in Wave Velocity in the Concentration Range of 0.3–0.5 a.f.

The properties of the Pd film are inserted in the model to simulate different levels of hydrogen concentrations. Figure 7 and Figure 8 show the phase profiles of the frequency response at the different concentration levels. The (⋆) sign on each phase profile illustrates the phase value of the center frequency. This is the phase value used in calculating the change in wave velocity. The normalized change in wave velocity (Δvv) is calculated using (Equation 18) and plotted in Figure 9.

**Figure 10.**
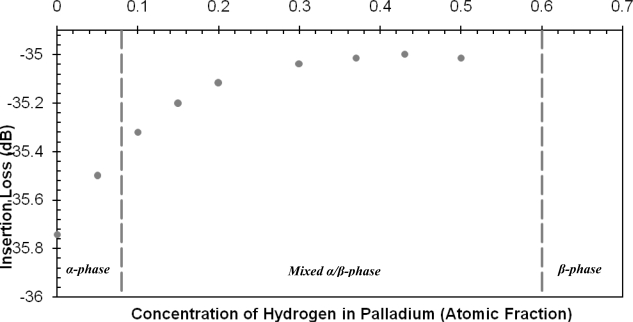
Insertion loss values at different levels of hydrogen concentrations.

The values are plotted with a negative sign to indicate a reduction in wave velocity. It can be seen that as more hydrogen is absorbed by the Pd film the velocity continues to decrease up to 0.5 a.f but the reduction in wave velocity is negligible in the concentration range of 0.3–0.5 a.f. In addition to the reduction in wave velocity the attenuation of the wave is monitored and plotted in Figure 10, which illustrates the insertion loss values due to hydrogen absorption. The insertion loss profile adopts a similar trend to the change in wave velocity, indicating that the attenuation of the wave in the 0.3–0.5 a.f region is almost constant. Table 7 lists the results of the SAW sensor at the different concentration levels.

## 7. Discussion

A finite element model of a SAW sensor is developed and tested with different levels of hydrogen concentrations. Hydrogen absorption deteriorates the lattice structure and softens the crystal as illustrated by the decreasing modulus of elasticity and density values in Table 6. According to pressure-composition isotherms [38] the lattice structure of palladium hydride at temperatures below 300 °C decomposes into an α-phase and a hydrogen rich β-phase. At room temperature the α-phase exists at a very low hydrogen concentration region; *c_H/Pd_* *< 0.008* a.f [38]. At a higher concentration of absorbed hydrogen the β-phase starts forming from the discontinuous expansion of the α-phase and is therefore highly distorted [38], which causes the significant reduction in the modulus of elasticity and density. The β-phase is completely formed at *c_H/Pd_ > 0.6* a.f. The change in the properties of the palladium hydride system leads to a reduction in SAW velocity.

The frequency response of the SAW sensor with a pure palladium film is shown in Figure 6. The insertion loss profile has a centre peak at the operating frequency of the sensor, which in this case is 128 MHz. In addition, the linear phase response of the sensor is also shown; the value at 128 MHz is used for calculating the change in phase as the concentration of absorbed hydrogen in the film increases. The phase profiles in Figure 7 and Figure 8 illustrate the change in phase at the centre frequency of the SAW sensor at different levels of hydrogen absorption. According to (Equation 16) this change implies a reduction in wave velocity with increased hydrogen concentration.

According to Figure 9 the velocity decrease up to a 0.3 a.f, then the reduction is of a much lower magnitude in the concentration region of 0.3–0.5 a.f. A similar behavior is reported by Jakubik *et al.* [11], who uses a YZ-LiNbO_3_ SAW sensor with bi-layer sensing film, composed of a 160 nm layer metal-free phthalocyanine and a 20 nm palladium layer. The experiment is set-up such that the SAW sensor operates in an oscillator configuration and is exposed to various concentrations (volume %) of hydrogen in air at room temperature to determine the sensor response below the explosive limit of 4%. The change in frequency of the sensor in an oscillator configuration is related to the change in velocity by [39];

(19) $$\left(\frac{\Delta v}{v}\right)=G\left(\frac{\Delta f}{f}\right)$$

where *G* is a constant.

**Figure 11.**
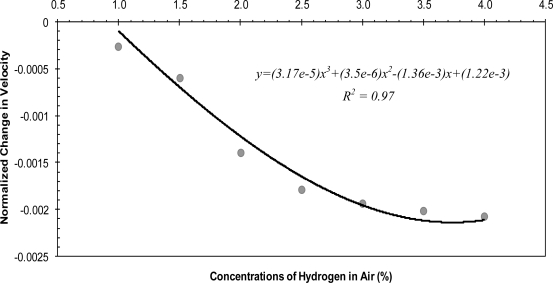
Normalized change in wave velocity at various H_2_ concentrations in air (volume %). Data is obtained from the frequency response values reported by Jakubik *et al.* [11].

According to the results reported by Jakubik *et al.* the increase in the magnitude of the change in oscillating frequency is due to the formation of the β-phase. The values of the normalized change in oscillating frequency from Jakubik *et al.* [11] have been converted to normalized change in wave velocity and plotted in Figure 11 for illustration.

The results of the current simulation in Figure 9 follow the same trend as that of Jakubik *et al.* The data points in both figures are fitted with a cubic polynomial function and in both cases the R^2^ value is above 0.97. The changing properties of the palladium film due to hydrogen absorption also affect the attenuation of the SAW wave. The insertion loss values in Figure 10 illustrate that as the hydrogen concentration increases in the film the insertion loss values increase accordingly indicating less attenuation. The insertion loss values adopt a similar behavior to the change in wave velocity since the values continue to increase but with a smaller magnitude in the concentration range of 0.3–0.5 a.f. This result is expected because as mentioned earlier the formation of the β-phase softens the crystal and as the β-phase dominates the lattice, the properties of the Pd film continue to change but with a lower magnitude. These results of the normalized velocity and insertion loss indicate that the magnitude of the response of the SAW sensor increases with increasing hydrogen absorption in the Pd film then the magnitude decreases as the β-phase of palladium hydride dominates the lattice structure.

## 8. Conclusions

A finite element model of a SAW sensor was developed and verified using simulation and experimental results from the literature. The sensor configuration was then changed to allow for hydrogen detection. A palladium film was added on the surface of a bare YZ-LiNbO_3_ substrate because Pd has a high affinity for hydrogen. A transient analysis was carried out from which the frequency response was determined. The phase response curves were compared for the different levels of hydrogen absorption and the change in phase with respect to the pure Pd case was calculated. The change in phase was used to calculate the normalized change in wave velocity for each case. Phase results indicated that with increased hydrogen absorption the wave velocity decreases. This behavior was found to be due to the formation of the β-phase of the palladium hydride structure, which causes distortions to the crystal and softens it. In addition, insertion loss values were used to determine the change in attenuation of the wave due to hydrogen absorption. Results illustrated that the softening of film as the β-phase dominates the lattice structure lead to a reduction in the magnitude of attenuation of the wave.

## Figures and Tables

**Table 1.** Material Properties for lithium niobate.

***Elastic Matrix In Stiffness Form (x10^11^Pa)***		***Piezoelectric Matrix at Constant Strain(C/m^2^)***		***Permittivity Matrix at Constant Strain (x10^−11^F/m)***	
*C_11_*	*2.03*	*e_15_*	*3.7*	*ε_11_*	*39*
*C_12_*	*0.573*	*e_22_*	*2.5*	*ε_33_*	*20.4*
*C_13_*	*0.752*	*e_31_*	*0.2*		
*C_14_*	*0.085*	*e_33_*	*1.3*		
*C_33_*	*2.424*				
*C_44_*	*0.595*				
*C_66_*	$\frac{c_{11}-c_{12}}{2}=0.7285$				

**Table 2.** Material Properties for zinc oxide.

***Elastic Matrix In Stiffness Form (x10****^11^****Pa)***		***Piezoelectric Matrix at Constant Strain(C/m****^2^****)***		***Permittivity Matrix at Constant Strain (x10****^−11^****F/m)***	
*C_11_*	*1.57*	*e_15_*	−*0.45*	*ε_11_*	*7.35*
*C_12_*	*0.89*	*e_31_*	−*0.51*	*ε_33_*	*7.79*
*C_13_*	*0.83*	*e_33_*	*1.22*		
*C_33_*	*2.08*				
*C_44_*	*0.38*				
*C_66_*	$\frac{c_{11}-c_{12}}{2}=0.34$				

**Table 3.** Comparing current simulation results with results published in the literature.

	**Center Frequency (MHz)**	**IL(dB)**

**Experimental [31]**	103	−34.3
**Simulation [31]**	100	−37.5
**Current Simulation**	100.56	−35.5

**Table 4.** Parameters of the YZ-LiNbO_3_ SAW sensor model.

**Parameter**	**Value**
Substrate Dimensions	3,500 × 400 × 500 μm
Number of Electrode Pairs for each IDT	12
Wavelength (λ)	26.5 μm
Electrode width and spacing between the electrodes	λ/4
Propagation Distance	523.2 μm
Electrode Length	200 μm
Dimensions of Pd Film	423 × 175 × 2 μm

**Table 5.** Thickness values for the palladium film at different levels of hydrogen concentrations.

c(HPd)	(ΔVV)	*m_H_* (g/mol)	*m_pd_* (g/mol)	Thickness [h(μm)]
0	0	1.008	106.42	2
0.05	0.0095	1.008	106.42	2.019
0.1	0.019	1.008	106.42	2.038
0.15	0.0285	1.008	106.42	2.057
0.2	0.038	1.008	106.42	2.076
0.3	0.057	1.008	106.42	2.114
0.37	0.0703	1.008	106.42	2.1406
0.43	0.0817	1.008	106.42	2.1634
0.5	0.095	1.008	106.42	2.19

**Table 6.** Material Properties for the palladium film at different hydrogen concentrations.

c(HPd)	*E*(*Gpa*)	*ρ_c_* (kg/m^3^)
0	128	12020
0.05	126.2	12019.98
0.1	124.5	12008.57
0.15	123	11985.84
0.2	121.36	11951.94
0.3	118.5	11851.5
0.37	116.83	11756.331
0.43	115	11659.352
0.5	113.73	11529.29

**Table 7.** Frequency response of the YZ-LiNbO_3_ SAW sensor at different hydrogen concentrations.

c(HPd)	*Phase* (*ϕ*) *in Deg.*	|Δϕ|	|Δϕϕ|=|Δvv|	*Frequency (MHz)*	*Insertion Loss (dB)*
Pure Pd	−12629	0.00	0.00	128.42	−35.743
0.05	−12646.8	17.8	0.00141	128.42	−35.5
0.1	−13023	394	0.0312	128.42	−35.32
0.15	−13395.8	766.8	0.0607	128.42	−35.202
0.2	−13408	779	0.0617	128.42	−35.12
0.3	−13735	1106	0.0876	127.93	−35.04
0.37	−13743	1114	0.0882	127.93	−35.014
0.43	−13748	1119	0.0886	127.93	−34.99
0.5	−13748	1119	0.0886	127.93	−35.01
